# Effects of probiotic supplementation on growth and meat quality of Simmental bulls

**DOI:** 10.3389/fvets.2025.1690922

**Published:** 2026-02-12

**Authors:** Yongqing Liu, Gaifang Wang, Rui Wang, Ying He, Xia Zhang, Caiping Feng

**Affiliations:** Department of Biology and Food Engineering, Lyuliang University, Lishi, China

**Keywords:** *lactobacillus*, growth, meat quality, intramuscular fat, bulls

## Abstract

Probiotics are well established to enhance animal immunity and gastrointestinal health, while also improving meat tenderness and antioxidant status. This study evaluated the effects of dietary probiotic supplementation on growth and meat quality of Simmental bulls. Forty Simmental bulls were randomly assigned by body weight to one of four groups, receiving a basal diet supplemented with probiotics at 0, 0.1, 0.2, or 0.3 g per kg of dry matter (DM). The trial included a 20-day adaptation phase followed by a 60-day experimental period. Dietary probiotic supplementation did not affect DM intake or carcass traits (*p* > 0.05) but linearly increased average daily gain (*p* = 0.001) and reduced the feed conversion ratio (*p* = 0.001). Increasing the probiotic dose linearly enhanced meat redness (*p* = 0.007), while decreasing cooking loss (*p* = 0.022) and shear force (*p* = 0.014). Intramuscular fat content and triglyceride levels increased significantly (*p* = 0.017 and 0.001, respectively), showing linear and quadratic patterns. Antioxidant indices were also strengthened with increasing dosage, including total antioxidant capacity (*p* = 0.005) and glutathione peroxidase activity (*p* = 0.012) in a linear manner, and catalase activity (*p* = 0.012) in a quadratic manner. The probiotic supplementation linearly reduced the proportions of C16:0 (*p* = 0.008) and saturated fatty acids (SFA) (*p* = 0.001), and increased the proportions of C18:1n9c (*p* = 0.013), C22:5n3 (*p* = 0.017), monounsaturated fatty acids (*p* = 0.009), polyunsaturated fatty acids (*p* = 0.007), unsaturated fatty acids (UFA) (*p* = 0.001), and the UFA/SFA ratio (*p* = 0.001). The probiotics linearly upregulated the mRNA expression of fatty acid synthase, acetyl-CoA carboxylase, and sterol regulatory element binding transcription factor 1 (*p* = 0.002, 0.001, and 0.010), quadratically upregulated stearoyl-CoA desaturase 1 and peroxisome proliferate-activated receptor *α* (*p* = 0.001 and 0.003), and linearly downregulated carnitine palmitoyltransferase 1B (*p* = 0.009). Collectively, our results establish that 0.2 g/kg DM is the optimal dosage of dietary probiotic supplementation for simultaneously enhancing bull growth and beef quality. This work validates probiotics as a sustainable feeding strategy and opens new avenues for improving meat quality through microbial manipulation.

## 1 Introduction

With growing awareness of healthy living, consumer demand for high-quality beef has significantly increased. However, intensive farming practices often improve feeding efficiency at the expense of beef quality (1, 2). Enhancing nutritional value and sensory attributes, particularly intramuscular fat (IMF) content and fatty acid composition, has therefore become a major goal in livestock production. IMF significantly influences beef quality, such as appearance, tenderness, water-holding capacity, and flavor (3). A balanced ratio of unsaturated fatty acids (UFA) in IMF also provides health benefits, including reduced risk of human chronic diseases (4). Understanding the mechanisms that regulate IMF deposition and fat metabolism is thus essential for breeding livestock with improved meat quality and mitigating metabolic disorders.

Probiotics are extensively utilized in animal production due to their beneficial effects on growth performance, immunity, and gastrointestinal health (5, 6). Emerging evidence indicates that probiotic supplementation can modulate meat quality and fat deposition in livestock (7). For example, dietary lactic acid bacteria has been shown to reduce pork shear force and alter fatty acid profiles through upregulation of genes like stearoyl-CoA desaturase (*SCD*) and peroxisome proliferate-activated receptor *α* (*PPARα*) (8). Similarly, lactic acid bacteria supplementation in Sunit lambs enhanced tenderness and IMF deposition by increasing lipogenic gene expression (acetyl-CoA carboxylase, *ACC*; sterol regulatory element binding transcription factor 1, *SREBF1*; fatty acid synthase, *FAS*) and suppressing lipolytic genes (protein kinase AMP-activated catalytic subunit α2, *AMPKα2*; carnitine palmitoyltransferase 1B, *CPT1B*) (9). These findings suggested that lactic acid bacteria improved meat quality by promoting lipogenesis and inhibiting lipolysis. However, few studies have examined its effects on IMF deposition and regulatory pathways in cattle.

We hypothesized that probiotic supplementation would enhance growth and IMF deposition through modulation of lipogenic pathways. This study investigated how different levels of probiotic supplementation affect growth and meat quality in bulls. To our knowledge, this is the first study assessing graded probiotic supplementation on IMF deposition and gene expression in Simmental bulls. The finding will offer important insights into improving beef quality through microbial strategies and support sustainable livestock production.

## 2 Materials and methods

The probiotic supplement (10^11^CFU/g), composed of a 1:1 blend of *Lacticaseibacillus casei* 29 and *Lactiplantibacillus plantarum* 194, was obtained from Animik Biotechnology Co., Ltd. (Luohe, China). This formulation incorporated a 0.5% poly-*γ*-glutamic acid coating to ensure bacterial viability during gastrointestinal transit.

### 2.1 Animals and experimental design

The experimental protocol received approval from the Animal Care and Use Committee of Lyuliang University and followed the guidelines in the Guide for the Care and Use of Agricultural Animals in Research and Teaching.^1^

Forty Simmental bulls (440 ± 18 days old, 562 ± 7.5 kg initial body weight, BW) were stratified by BW and randomly allocated to one of four dietary treatments (n = 10 per group) in a completely randomized design. Throughout the trial, each animal was housed individually in separate pens. The control group (CON) received a basal diet without probiotics, while the treatment groups received graded levels of probiotic supplementation: low-dose (LP, 0.1 g/kg DM), medium-dose (MP, 0.2 g/kg DM), and high-dose (HP, 0.3 g/kg DM). These supplementation levels were selected based on a previous study by He et al. (7). The total mixed ration was formulated to meet the nutritional requirements for beef cattle as established in the Nutrient Requirements of Beef Cattle (8th revised edition, NRC, 2016) (10), with detailed diet composition and nutrient levels provided in Table 1. Probiotics were premixed into approximately 150 g of the daily ration before feeding to ensure uniform distribution. The experiment spanned 80 days, including a 20-day adaptation phase followed by a 60-day experimental period. Treatments were initiated at the beginning of adaptation. All bulls were fed twice daily (07:00 and 19:00 h) and had unrestricted access to feed and water.

**Table 1:** Ingredient and chemical composition of experimental diets (DM basis).

Items	Contents [g/kg]			
	Control	LP	MP	HP
Ingredients				
Corn silage	500	500	500	500
Corn grain, ground	275	275	275	275
Wheat bran	34	34	34	34
Soybean meal	120	120	120	120
Cottonseed meal	40	40	40	40
Probiotics	0	0.1	0.2	0.3
Calcium carbonate	5	5	5	5
Salt	5	5	5	5
Calcium biphosphate	15	15	15	15
Sodium bicarbonate	5	5	5	5
Mineral and vitamin premix^a^	1	1	1	1
Chemical composition				
NEm (MJ/kg)	7.56	7.56	7.56	7.56
NEg (MJ/kg)	5.17	5.17	5.17	5.17
Dry matter	605.3	605.3	605.3	605.3
Organic matter	940.1	940.1	940.1	940.1
Ether extract	52.4	52.4	52.4	52.4
Crude protein	155.5	155.5	155.5	155.5
Neutral detergent fiber	322.8	322.8	322.8	322.8
Acid detergent fiber	179.0	179.0	179.0	179.0
Calcium	6.7	6.7	6.7	6.7
Phosphorus	4.2	4.2	4.2	4.2

a Contained per kg premix: 100 mg Co, 8,500 mg Cu, 50,000 mg Fe, 30,000 mg Mn, 30,000 mg Zn, 300 mg I, 300 mg Se, 7,500,000 IU vitamin A, 1200, 000 IU vitamin D, and 40, 000 IU vitamin E.

### 2.2 Growth performance, slaughter procedures and sample collection

Body weight was recorded on two consecutive days at the start (day 1) and end (day 60) of the trial period before the morning feeding. Daily dry matter intake (DMI) was determined by subtracting orts from the feed provided. Feed conversion ratio (FCR) was calculated as total DMI divided by average daily gain (ADG).

On day 61, after a 12-h fast, five bulls per treatment group were randomly selected, transported to a slaughterhouse located 5 km from the farm, weighed, and humanely slaughtered according to standard halal procedures (11). Carcass weight was recorded immediately, and dressing percentage was calculated as carcass weight divided by live weight. Carcasses were chilled at 4 °C for 24 h before dissection. The loin-eye area was measured between the 12th and 13th ribs with a planimeter. Backfat thickness was determined with a digital caliper, positioned 4 cm from the carcass midline and 4 cm caudal to the last rib (12). Net meat weight was obtained by subtracting bone weight from carcass weight, and net meat percentage was derived as net meat weight divided by live weight.

A sample of approximately 10 g of longissimus thoracis (LT) was excised from the 12th rib region of the left side of the carcass, rapidly frozen in liquid nitrogen, and stored at −80 °C for subsequent fatty acid and gene expression analyses. Additional LT sections (approximately 500 g) spanning the 10th to 13th ribs were collected for meat quality and chemical composition assessment.

### 2.3 Meat quality

Muscle pH was measured at 45 min and 24 h post-mortem with a calibrated TESTO 205PH portable pH meter (TESTO Instruments, Germany) by inserting the probe at three standardized sites on the LT. To ensure the accuracy of the result, the mean value of three measurements for each sample was recorded at 25 °C. Meat color was evaluated at 24 h post-mortem using a Minolta CR-400 chromameter (Konica Minolta Sensing Inc., Japan), which was calibrated daily against a CR-A44 white reference plate under CLE LAB color space settings with a D65 illuminant, 2°standard observer, and 8 mm aperture (13). Three measurements per sample were averaged after rotating the probe 90°between each reading. For drip loss assessment, approximately 55 g of LT muscle was trimmed into 3.0 cm^3^ cubes, weighed (W_1_) at 24 h post-mortem, held in airtight bags at 4 °C for 24 h, and reweighed (W_2_). Drip loss was calculated as [(W_1 −_ W_2_)/W_1_] × 100 (12). Cooking loss was determined by weighing (M_1_) a 100 g LT sample (6 × 6 × 4 cm), heating it in an 80 °C water bath until the internal temperature reached 70 °C, cooling and drying it, and then reweighing (M_2_). The mean of three measurements per sample was used to compute cooking loss as [(M_1 −_ M_2_)/M_1_] × 100 (14). Shear force was analyzed according to Yu et al. (15) by cutting cooked samples into ten cuboids (1 × 1 × 3 cm) aligned with the muscle fiber and shearing them perpendicularly using a C-LT3B texture analyzer (Tenovo, China) equipped with a Warner-Bratzler blade, a 15 kg load cell, and a crosshead speed of 200 mm/min. The result represented the average of ten replicates.

Proximate composition of the LT muscle, including IMF, moisture, ash, and crude protein, was determined following AOAC (2000) guidelines (16). LT samples were homogenized using a Philips HR2657 grinder (Eindhoven, Netherlands), lyophilized, and pulverized. Lipids were extracted from the powder with petroleum ether using a Soxtec 2055 system (Foss Tecator AB, Höganäs, Sweden) for IMF quantification. Muscle triglyceride (TG) content was quantified using the GPO-PAP enzymatic assay (Nanjing Jiancheng Bioengineering Institute, China).

### 2.4 Antioxidant status

Antioxidant status was assessed by homogenizing 0.1 g of LT tissue in 0.9% saline (1:9, w/v) at 1,500 rpm for 2 min, centrifuging the homogenate at 3,500×*g* for 10 min at 4 °C, and analyzing the supernatant for total antioxidant capacity (T-AOC), malondialdehyde (MDA), catalase (CAT), glutathione peroxidase (GSH-Px), and superoxide dismutase (SOD) using commercial kits (Nanjing Jiancheng Bioengineering Institute) and a SpectraMax M5 microplate reader (Molecular Devices, USA).

### 2.5 Fatty acids profile

Fatty acid profiles were analyzed using a previously described method (17). Each 10 g sample was homogenized with 2 mL of an internal standard solution containing methyl heptadecanoate (1 mg/mL) and butylated hydroxytoluene (2 mg/mL), followed by methylation. After the reaction, fatty acid methyl esters (FAMEs) were extracted using n-hexane, and the upper organic phase was collected. Fatty acid concentrations were quantified via gas chromatography–mass spectrometry (GC2010 Plus GC–MS, Shimadzu Scientific Instruments, USA) based on the peak area of the internal standards. Fatty acid composition was identified by comparing retention times against those of FAME standards (CRM47885, Sigma, Darmstadt, Germany). The temperature program was configured as follows: injector temperature, 230 °C; detector temperature, 230 °C; initial column temperature, 55 °C held for 0.5 min, increased to 205 °C at 35 °C/min and held for 3 min, then raised to 230 °C at 5 °C/min and maintained for 2.5 min; injection volume, 1 μL; split ratio, 60:1; carrier gas, nitrogen at a flow rate of 1 mL/min. Results were expressed as the relative percentage of each fatty acid in the total IMF.

### 2.6 Quantitative real-time PCR analysis

Quantitative real-time PCR (RT–qPCR) was performed according to established protocols (18). Total RNA was extracted from LT muscle using TRIzol reagent (Invitrogen, Carlsbad, CA, USA), and RNA integrity was confirmed by agarose gel electrophoresis. Qualified RNA samples (1 μg) were reverse transcribed into cDNA using a PrimeScript RT Reagent Kit (Takara Biotechnology, Dalian, China). Quantitative PCR amplification was conducted in duplicate on an ABI QuantStudio™ RT–PCR system (Thermo Fisher Scientific, Waltham, MA, USA). Primer sequences for target genes are listed in Table 2, and *β*-actin was used as the reference gene. The PCR amplification protocol followed established parameters (19). Relative gene expression was determined using the 2^−ΔΔCT^ method.

**Table 2:** PCR primers for real-time quantitative PCR assays.

Gene name	Primer sequence (5′–3′)	GenBank accession no.	Size (bp)
*β-actin*	F: CTCACGGAGCGTGGCTACA R: GCCATCTCCTGCTCGAGGTC	NM_001009784.3	107
*FAS*	F: GTGTGGTACAGCCCCTCAAG R: ACGCACCTGAATGACCACTT	XM_027974304.3	110
*ACC*	F: GACACATCACATCCGTCCTCT R: GTCCATCACCACAGCCTTCAT	XM_060394703.1	188
*SCD1*	F: TCCGACCTAAGAGCCGAGAA R: AGCACAACAACAGGACACCA	NM_173959.4	73
*DGAT1*	F: GCCCAAGGGTCTGAATGTGT R: CCTCCTTCCCACAGCATGG	XM_059893129.1	120
*LPL*	F: CCCGGCTTTGATATTGGGAAG R: TCAGGGACTTGTCATGGCATT	NM_001075120.1	142
*HSL*	F: AGAGTGCTTCTAGTCCTACTGC R: GACACGGTGAAGCAGAGGTTC	NM_001080220.1	117
*CPT1B*	F: TGTTCAACACCACTCGCATC R: CTCGTAGAGCCACAGCTTGA	XM_060411597.1	116
*FABP4*	F: AGATGGTGCTGGAATGTGTCA R: GGAGTTCGATGCAAACGTCA	NM_174314.2	103
*SREBF1*	F: CGCAAAGCCATCGACTACATC R: TGAGCTTCTGGTTGCTGTGCT	XM_027974786.2	52
*PPARα*	F: GGCCCCAGGTGGTGGA R: CCGGCCACAGACTGTTACTT	NM_001034036.1	123
*AMPKα2*	F: ATGAGGTGGTGGAGCAGAGG R: CGTGAGAGAGCCAGAGAGTGAA	NM_001112816.1	131

*FAS*, fatty acid synthase; *ACC*, acetyl-CoA carboxylase; *SCD1*, stearoyl-CoA desaturase 1; *DGAT1*, diacylglycerol O-acyltransferase homolog 1; *LPL*, lipoprotein lipase; *HSL*, hormone sensitive lipase; *CPT1B*, carnitine palmitoyltransferase 1B; *FABP4*, fatty acid binding protein 4; *SREBF1*, sterol regulatory element binding transcription factor 1; *PPARα*, peroxisome proliferate-activated receptor α; *AMPKα2*, protein kinase AMP-activated catalytic subunit α2.

### 2.7 Calculations and statistical analyses

Data were analyzed using SAS software (SAS Institute Inc., Cary, NC, USA) under a completely randomized experimental design (20). One-way ANOVA was applied to evaluate linear and quadratic responses to probiotic supplementation levels. The model was specified as Y_ij_ = *μ* + F_i_ + ε_ij_, where Y_ij_ represents the dependent variable, μ the overall mean, F_i_ the fixed effect of probiotic supplementation (i = 0, 0.1, 0.2, and 0.3 g probiotics/kg DM), and ε_ij_ the residual error. Orthogonal polynomial contrasts were utilized to characterize dose–response trends. *Post hoc* analyses were performed using Duncan’s multiple range test. Each bull was considered an experimental unit. Data are presented as mean ±standard error of the mean (SEM), with statistical significance set as *p* < 0.05.

## 3 Results

### 3.1 Growth performance and carcass traits

As shown in Table 3, dietary probiotic supplementation had no impact on the DMI of bulls (*p* > 0.05). Initial BW was similar across all treatment groups (*p* > 0.05). Both final BW and ADG increased linearly (*p* = 0.001 and 0.001) with probiotic supplementation. ADG was 16.5% greater in the MP group compared to the control (*p* = 0.003). FCR decreased linearly (*p* = 0.001) and was 13.9% lower in the MP group than in the control (*p* = 0.005). As presented in Table 4, dietary probiotic supplementation did not affect carcass traits (*p* > 0.05).

**Table 3:** Effects of probiotics on growth performance of bulls (*n* = 40).

Item	Treatment^1^				SEM	*p*-values		
	Control	LP	MP	HP		Treatment	Linear	Quadratic
DMI (kg/d)	12.20	12.22	12.29	12.32	0.071	0.128	0.052	0.990
Body weight (kg)								
0 d	563.2	561.6	562.0	564.1	1.155	0.371	0.532	0.101
60 d	628.6^b^	631.4^b^	638.5^a^	640.2^a^	2.130	0.001	0.001	0.767
ADG (kg/d)	1.09^b^	1.16^ab^	1.27^a^	1.26^a^	0.390	0.003	0.001	0.286
FCR (kg/kg)	11.29^a^	10.61^ab^	9.72^b^	9.80^b^	0.367	0.005	0.001	0.263

1 Control, LP, MP, and HP groups contained, respectively, 0, 0.1, 0.2 and 0.3 g probiotics/kg DM.

Different superscript letters in the same row differed significantly.

**Table 4:** Effects of probiotics on carcass traits of bulls (*n* = 20).

Item	Treatment^1^				SEM	*p*-values		
	Control	LP	MP	HP		Treatment	Linear	Quadratic
Live weight (kg)	616.0	616.9	619.3	618.4	3.827	0.828	0.446	0.729
Carcass weight (kg)	318.7	324.5	325.8	323.8	7.069	0.824	0.524	0.499
Carcass yield (%)	51.73	52.60	52.60	52.36	1.076	0.864	0.618	0.514
Backfat thickness (mm)	14.16	14.19	14.34	14.27	0.183	0.802	0.448	0.755
Loin eye area (cm^2^)	70.50	71.38	72.50	72.22	1.297	0.677	0.280	0.650
Net meat weight (kg)	288.2	293.5	294.0	288.0	7.285	0.781	0.997	0.314
Net meat rate (%)	46.79	47.56	47.47	46.57	1.197	0.798	0.848	0.337

1 Control, LP, MP and HP groups contained, respectively, 0, 0.1, 0.2 and 0.3 g probiotics/kg DM.

Different superscript letters in the same row differed significantly.

### 3.2 Meat quality

As shown in Table 5, pH did not differ among the treatment groups (*p* > 0.05). The a* value increased linearly (*p* = 0.007) with probiotic supplementation and was 3.1% higher in the MP group relative to the control (*p* = 0.043). Probiotic supplementation did not affect b*, L* values, or drip loss (*p* > 0.05). Cooking loss decreased linearly (*p* = 0.022) and was 6.9% lower in the MP group than in the control (*p* = 0.049). Shear force declined linearly (*p* = 0.014) and was 6.7% smaller in the MP group than in the control group (*p* = 0.040).

**Table 5:** Effects of probiotics on beef quality (*n* = 20).

Item	Treatment^1^				SEM	*p*-values		
	Control	LP	MP	HP		Treatment	Linear	Quadratic
pH_45min_	6.46	6.43	6.40	6.41	0.062	0.838	0.449	0.694
pH_24h_	5.62	5.59	5.57	5.58	0.058	0.878	0.508	0.661
a*	14.34^b^	14.53^ab^	14.79^a^	14.83^a^	0.157	0.043	0.007	0.566
b*	9.65	9.58	9.59	9.57	0.326	0.996	0.844	0.925
L*	36.25	36.98	37.40	37.52	1.256	0.798	0.352	0.758
Drip loss (%)	3.83	3.70	3.71	3.69	0.237	0.943	0.627	0.777
Cooking loss (%)	26.12^a^	24.92^ab^	24.31^b^	24.68^b^	0.533	0.049	0.022	0.089
Shear force (N)	59.27^a^	56.39^b^	55.32^b^	55.70^b^	1.107	0.040	0.014	0.108
Moisture (%)	72.79	72.22	71.43	71.40	0.573	0.223	0.051	0.614
Crude protein (%)	20.58	20.95	21.10	21.12	0.240	0.246	0.068	0.403
Ash (%)	1.12	1.09	1.10	1.10	0.055	0.971	0.854	0.718
IMF (%)	4.05^b^	4.24^b^	4.59^a^	4.31^b^	0.120	0.004	0.010	0.017
TG (mmol/gprot)	0.40^b^	0.42^b^	0.48^a^	0.46^a^	0.013	0.001	0.001	0.055

1 Control, LP, MP and HP groups contained, respectively, 0, 0.1, 0.2 and 0.3 g probiotics/kg DM.

Different superscript letters in the same row differed significantly.a*, meat redness; b*, meat yellowness; L*, meat lightness.

Moisture, crude protein, and ash contents were similar across groups (*p* > 0.05). IMF increased quadratically (*p* = 0.017) and was 13.3% higher in the MP group than in the control group (*p* = 0.004). TG increased linearly (*p* = 0.001) and was greater in the MP and HP groups than in the others (*p* = 0.001).

### 3.3 Antioxidant status

As shown in Table 6, T-AOC and GSH-Px activity increased linearly (*p* = 0.005 and 0.012) and were 46.6 and 6.5% higher in the MP group compared to the control (*p* = 0.023 and 0.049). Probiotic supplementation did not affect MDA or SOD (*p* > 0.05). CAT activity increased quadratically (*p* = 0.012) and was 22.5% higher in the MP group than in the control group (*p* = 0.001).

**Table 6:** Effects of probiotics on antioxidant status of *Longissimus thoracis* in bulls (*n* = 20).

Item	Treatment^1^				SEM	*p*-values		
	Control	LP	MP	HP		Treatment	Linear	Quadratic
T-AOC (U/mgprot)	0.30^b^	0.38^ab^	0.44^a^	0.43^a^	0.039	0.023	0.005	0.173
MDA (μmol/gprot)	2.44	2.34	2.23	2.20	0.111	0.276	0.062	0.683
SOD (U/mgprot)	43.84	44.34	44.60	44.63	1.220	0.920	0.526	0.798
GSH-Px (U/mgprot)	33.21^b^	34.89^ab^	35.39^a^	35.53^a^	0.850	0.049	0.012	0.214
CAT (U/gprot)	6.08^c^	6.58^bc^	7.45^a^	6.83^b^	0.248	0.001	0.003	0.012

1 Control, LP, MP and HP groups contained, respectively, 0, 0.1, 0.2 and 0.3 g probiotics/kg DM.

Different superscript letters in the same row differed significantly.

### 3.4 Fatty acids

As shown in Table 7, the saturated fatty acids (SFA) content in beef from the MP and HP groups was significantly lower than in the control group (*p* = 0.001). Increasing the level of probiotics led to a linear reduction in SFA content (*p* = 0.001). The content of C16:0 was also markedly lower in the MP and HP groups compared to the control (*p* = 0.034), and decreased linearly with increasing probiotic supplementation (*p* = 0.008).

**Table 7:** Effects of probiotics on fatty acid profiles of *Longissimus thoracis* (*n* = 20) (g/100 g fatty acids).

Item	Treatment^1^				SEM	*p*-values		
	Control	LP	MP	HP		Treatment	Linear	Quadratic
C12:0	1.16	1.17	1.14	1.15	0.042	0.907	0.671	0.975
C14:0	2.41	2.41	2.42	2.42	0.166	0.999	0.930	0.999
C15:0	0.26	0.25	0.19	0.21	0.028	0.204	0.081	0.585
C16:0	24.62^a^	23.70^ab^	21.86^b^	22.20^b^	0.783	0.034	0.008	0.364
C16:1	2.78	2.85	2.88	2.90	0.231	0.970	0.850	0.672
C17:0	0.98	0.77	0.86	0.98	0.137	0.389	0.834	0.110
C17:1	0.76	0.68	0.80	0.78	0.058	0.194	0.286	0.508
C18:0	16.87	16.46	16.24	16.19	0.487	0.484	0.151	0.595
C18:1n9c	35.77^b^	36.80^ab^	38.17^a^	37.79^a^	0.661	0.045	0.013	0.230
C18:1n9t	1.42	1.39	1.34	1.37	0.161	0.734	0.281	0.836
C18:2	3.18	3.51	3.77	3.73	0.223	0.121	0.056	0.395
C18:3n6c	1.29	1.36	1.45	1.38	0.163	0.684	0.241	0.895
C20:0	0.10	0.11	0.11	0.10	0.017	0.985	0.999	0.704
C20:1	0.28	0.26	0.29	0.30	0.039	0.819	0.577	0.586
C20:2	1.17	1.16	1.16	1.17	0.160	0.984	0.806	0.776
C20:3n6c	3.42	3.49	3.63	3.65	0.150	0.396	0.106	0.798
C20:4	2.12	2.08	2.11	2.09	0.098	0.966	0.862	0.645
C22:0	0.15	0.12	0.12	0.12	0.016	0.460	0.221	0.380
C22:5n3	0.38^b^	0.51^ab^	0.58^a^	0.55^a^	0.062	0.048	0.017	0.117
C22:6n3	0.64	0.69	0.69	0.68	0.090	0.967	0.758	0.721
C24:0	0.25	0.23	0.19	0.24	0.032	0.425	0.589	0.208
ω6PUFA	4.72	4.86	5.08	5.13	0.224	0.433	0.115	0.840
ω3PUFA	1.03	1.20	1.26	1.23	0.120	0.233	0.095	0.224
ω6/ω3	4.67	4.19	4.11	4.25	0.411	0.729	0.434	0.429
SFA	46.78^a^	45.22^ab^	43.13^c^	43.62^bc^	0.697	0.001	0.001	0.091
UFA	53.22^c^	54.78^bc^	56.87^a^	56.38^ab^	0.697	0.001	0.001	0.091
UFA/SFA	1.14^b^	1.21^b^	1.32^a^	1.30^a^	0.036	0.001	0.001	0.120
MUFA	41.00^b^	41.97^ab^	43.50^a^	42.91^a^	0.619	0.026	0.009	0.171
PUFA	12.22^b^	12.81^ab^	13.36^a^	13.47^a^	0.399	0.044	0.007	0.453
PUFA/MUFA	0.298	0.306	0.306	0.316	0.012	0.624	0.222	0.918

1 Control, LP, MP and HP groups contained, respectively, 0, 0.1, 0.2 and 0.3 g probiotics/kg DM.

SFA was saturated fatty acid; UFA was unsaturated fatty acid; MUFA was monounsaturated fatty acids; PUFA was polyunsaturated fatty acid. ω3PUFA = C22:5n3 + C22:6n3; ω6PUFA = C18:3n6c + C20:3n6c; ω9PUFA = C18:1n9c + C18:1n9t. Different superscript letters in the same row differed significantly.

The monounsaturated fatty acids (MUFA) content was significantly higher in the MP and HP groups than in the control (*p* = 0.026), and increased linearly with probiotic level (*p* = 0.009). In particular, C18:1n9c was elevated in the MP and HP groups relative to the control (*p* = 0.045), and exhibited a linear upward trend with increasing probiotics (*p* = 0.013). Similarly, polyunsaturated fatty acids (PUFA) content was higher in the MP and HP groups than in the control (*p* = 0.044), and increased linearly with probiotic supplementation (*p* = 0.007). Notably, C22:5n3 was also higher in the MP and HP groups compared to the control (*p* = 0.048), and rose linearly with probiotic level (*p* = 0.017). Overall, the UFA/SFA ratio was significantly greater in the MP and HP groups than in the other groups (*p* = 0.001).

### 3.5 The expression of lipid metabolism related genes

As shown in Table 8, the relative expression of *FAS*, *ACC*, and *SREBF1* in the MP group was significantly increased by 76.2, 39.5 and 57.4%, compared to the control (*p* = 0.010, 0.001, and 0.021, respectively). Among all experimental groups, the MP group showed the highest expression of *SCD1* and *PPARα,* with 1.74-fold and 0.32-fold upregulation relative to the control (*p* = 0.001 and 0.002). In contrast, *CPT1B* expression was the lowest in the MP group, showing a 0.29-fold downregulation compared to the control (*p* = 0.009). Increasing probiotic levels produced a linear upregulation in *FAS*, *ACC*, and *SREBF1* expression (*p* = 0.002, 0.001, and 0.010), a quadratic upregulation in *SCD1* and *PPARα* (*p* = 0.001 and 0.003), and a linear downregulation in *CPT1B* (*p* = 0.009).

**Table 8:** Effects of probiotics on genes expression related to lipid metabolism in the *Longissimus thoracis* of bulls (*n* = 20).

Item	Treatment^1^				SEM	*p*-values		
	Control	LP	MP	HP		Treatment	Linear	Quadratic
*FAS*	1.26^b^	1.68^ab^	2.22^a^	2.10^a^	0.284	0.010	0.002	0.177
*ACC*	1.52^b^	1.72^b^	2.12^a^	2.10^a^	0.123	0.001	0.001	0.303
*SCD1*	8.27^d^	11.52^c^	22.71^a^	17.81^b^	1.269	0.001	0.001	0.001
*DGAT1*	6.21	6.59	6.66	6.72	1.100	0.971	0.668	0.847
*LPL*	14.02	13.62	10.58	12.72	1.688	0.324	0.275	0.368
*HSL*	0.37	0.36	0.34	0.37	0.058	0.956	0.881	0.640
*CPT1B*	23.06^a^	22.64^a^	16.46^b^	19.23^ab^	1.890	0.009	0.009	0.249
*FABP4*	2.70	2.77	3.39	3.54	0.462	0.218	0.051	0.894
*SREBF1*	27.46^b^	37.13^ab^	43.23^a^	39.76^a^	4.610	0.021	0.010	0.062
*PPARα*	9.57^c^	11.25^ab^	12.61^a^	11.06^b^	0.652	0.002	0.011	0.003
*AMPKα2*	0.96	0.92	0.87	0.90	0.089	0.760	0.408	0.582

1 Control, LP, MP and HP groups contained, respectively, 0, 0.1, 0.2 and 0.3 g probiotics/kg DM.

*FAS*, fatty acid synthase; *ACC*, acetyl-CoA carboxylase; *SCD1*, stearoyl-CoA desaturase 1; *DGAT1*, diacylglycerol O-acyltransferase homolog 1; *LPL*, lipoprotein lipase; *HSL*, hormone sensitive lipase; *CPT1B*, carnitine palmitoyltransferase 1B; *FABP4*, fatty acid binding protein 4; *SREBF1*, sterol regulatory element binding transcription factor 1; *PPARα*, peroxisome proliferate-activated receptor α; *AMPKα2*, protein kinase AMP-activated catalytic subunit α2.

Different superscript letters in the same row differed significantly.

## 4 Discussion

### 4.1 Growth performance and carcass traits

Dietary supplementation with probiotics did not affect DMI in bulls, a result consistent with the findings of Zhang et al. (21). The observed increase in ADG, however, likely originated from probiotic-induced modulation of the gut microbiota, which enhanced nutrient digestion, absorption, and muscle metabolism. Supporting this mechanism, Jiang et al. (22) found that *L. plantarum* 299v supplementation increased fecal microbial diversity in preweaning calves, suggesting a pathway for improved growth performance. Further evidence indicated that the rumen microbiota supported bull growth by optimizing nutrient digestion, while lactic acid bacteria species improve intestinal nutrient absorption through villus elongation and functional maturation (19, 23). Additionally, emerging research highlighted the role of gut microbiota in regulating muscle metabolism via the gut-muscle axis (24). Short-chain fatty acids (SCFAs) modulate glucose metabolism by binding to free fatty acid receptor (FFAR1)/FFAR4 and stimulating glucagon-like peptide-1 (GLP-1), which regulates insulin and glucagon secretion. Gut microbes also convert primary to secondary bile acids, activating G protein coupled receptor for bile acids (TGR5) to maintain energy homeostasis. Meanwhile, intestinal genes including fasting induced adipose factor and *PPARs* regulate lipid metabolism by mediating fatty acid uptake and mitochondrial *β*-oxidation. Microbiota modulation improves systemic physiology, thereby indirectly supporting muscle development (25). For instance, supplementing bulls with *Limosilactobacillus mucosae* CRL2069 throughout a 104-day fattening cycle significantly improved growth performance (5). In contrast, carcass traits remained unaffected by probiotic supplementation, aligning with observations by Hernández-García et al. (26), who reported no significant effect of probiotics on carcass yield in goats. The reasons may be relation to limited trial duration and age factor. These findings collectively demonstrate that probiotics improve growth performance without modifying carcass characteristics.

### 4.2 Meat quality

Meat pH values were unaffected by probiotic supplementation and remained within the acceptable range of 5.5–5.9, confirming that treatment did not compromise meat quality. Similarly, meat color measurements, including lightness (L* = 30–45), redness (a* = 10–25), and yellowness (b* = 5–15), fell within consumer-accepted thresholds. Notably, dietary probiotic supplementation significantly increased the a* value of the LT muscle in bulls, indicating improved redness without affecting other color parameters. This enhancement in muscle redness may be attributed to the antioxidant properties of lactic acid bacteria. Meat color is primarily determined by the redox state of myoglobin: oxygen-bound oxymyoglobin (OxyMb) imparts a bright red color, whereas oxidation to metmyoglobin (MetMb) causes undesirable browning (27). Lactic acid bacteria likely reduces myoglobin oxidation through several mechanisms, including free radical scavenging, metal ion chelation, and upregulation of endogenous antioxidant enzymes (28). By inhibiting MetMb formation and promoting OxyMb stability, probiotic supplementation helped to preserve meat redness, an effect also reported in lactic acid bacteria-treated Sunit lamb (29). Water-holding capacity, reflected by drip loss and cooking loss, is largely influenced by muscle protein integrity and IMF content. Higher IMF levels correlated negatively with cooking loss, suggesting that lipid deposits help to preserve muscle structure (30). Shear force values remained below 60 N in all samples, meeting standards for fresh meat. Tenderness is influenced by factors such as pH, collagen cross-linking, fiber type distribution, and IMF content, with IMF contributing to tenderness by disrupting perimysial collagen networks (31). In this study, both shear force and cooking loss decreased following probiotic supplementation, likely due to changes in IMF deposition. Recent studies have shown that probiotic supplementation significantly reduced cooking loss and shear force in Sunit lamb (32).

Dietary supplementation with probiotics significantly increased IMF content in beef, likely by stimulating adipocyte activity and promoting fat deposition within skeletal muscle (33). This effect aligned with findings in Sunit lambs, where long-term supplementation with *L. casei* HM-09 and *L. plantarum* HM-10 also accelerated IMF accumulation (34). Probiotic supplementation also raised TG concentrations, further indicating enhanced lipid deposition. In ruminants, lactic acid bacteria species boost the production of short-chain fatty acids and facilitate fatty acid absorption into muscle via blood circulation (9). These findings suggest that probiotics modify carcass fat distribution by altering lipid metabolism. However, IMF and TG content did not increase further in the HP group, as excessive probiotic intake can disrupt the intestinal mucosal environment, lower intestinal pH, and impair digestive function, ultimately hindering lipid absorption (9).

### 4.3 Antioxidant status

Improvements in meat quality traits, including redness (a* value), and water-holding capacity, coincided with elevated T-AOC, GSH-Px, and CAT. Lipids and oxygen interact through free-radical chain reactions to form peroxides, but the antioxidant system mitigates oxidative damage by scavenging free radicals and decelerating oxidation (35). Probiotics such as lactic acid bacteria serve as natural antioxidants against reactive oxygen species via enzymatic and nonenzymatic mechanisms (36–38). Tang et al. (39) demonstrated the *in vitro* antioxidant potential of *L. plantarum*, and *in vivo* studies showed that probiotics raise T-AOC, SOD, and GSH-Px levels while reducing MDA content in mice (40). Notably, the dose–response behavior of IMF and TG to probiotics mirrored that of CAT activity. These findings lead us to conclude that moderate supplementation strengthens CAT-mediated protection of IMF, whereas excessive intake disrupts lipid homeostasis and promotes oxidative stress.

### 4.4 Fatty acids

Fatty acids constitute the primary component of IMF in beef and are key indicators of nutritional quality. SFA, particularly C16:0 and C18:0, which comprise roughly 40% of total fatty acids in beef, are linked to elevated risks of chronic diseases in humans. In contrast, UFA help to prevent the occurrence of cardiovascular diseases (41). Probiotic supplementation reduced the proportion of C16:0 and increased levels of C18:1n9c and C22:5n3 in beef, indicating that probiotics modulate fatty acid profiles to improve both tenderness and health benefits. Metabolites from lactic acid bacteria may inhibit ruminal biohydrogenation of dietary UFA, promoting their direct incorporation into muscle tissue (42). Probiotics can also upregulate SCD1 activity, thereby promoting the conversion of SFA to UFA (43). A recent study confirmed that probiotic supplementation increased MUFA content and the MUFA/SFA ratio in lamb meat (44).

### 4.5 The expression of lipid metabolism related genes

Probiotic supplementation upregulated lipogenic genes (*FAS*, *ACC*, *SREBF1*) and downregulated *CPT1B* expression, correlating with increased IMF and TG deposition. Probiotics modulate the gut microbiota, which in turn regulates lipid metabolism-related genes and subsequently influences fat content in skeletal muscle (7). In contrast, the absence of gut microbiota enhances fatty acid catabolism through elevated CPT1 activity. Therefore, appropriate supplementation with probiotics generally promotes IMF deposition. Lactic acid bacteria enhanced cellular lipid storage by upregulating *ACC* and downregulating *CPT1B* (45). By inhibiting AMPKα2 signaling, probiotics promoted expression of downstream targets *SREBF1*, *FASN*, and *ACC*, suppress *CPT1B* expression, and ultimately increase IMF deposition in the Sunit lambs (9). These results indicated that probiotics maintained IMF content by elevating lipogenesis and reducing lipolysis.

Furthermore, probiotics elevated *SCD1* and *PPARα* mRNA levels, which coincided with increased UFA content in beef. PPARα activation stimulated fatty acid biosynthetic pathways. The desaturase SCD1 significantly influenced fatty acid metabolism by controlling *de novo* MUFA synthesis in muscle (43). Probiotics also regulated fatty acid metabolism in pig muscle by inducing *SCD1* expression through *PPARα*-mediated signaling (8).

## 5 Conclusion

Dietary probiotic supplementation (0.2 g/kg DM) improved growth performance without affecting carcass traits. Notably, it enhanced beef redness and antioxidant capacity while reducing cooking loss and shear force. Probiotics also increased IMF content by upregulating lipogenic genes and suppressing lipolytic genes. Moreover, the observed shift in UFA profile could potentially involve probiotic-induced activation of the PPARα/SCD1 pathway. These findings support the use of probiotics as a sustainable strategy for quality beef production. Future studies should focus on evaluating long-term efficacy across different cattle breeds and production systems to facilitate broader application.

## Data Availability

The original contributions presented in the study are included in the article/Supplementary material, further inquiries can be directed to the corresponding author.

## Footnotes

1
https://www.asas.org/services/ag-guide
